# Team Cognition in Sport: How Current Insights Into How Teamwork Is Achieved in Naturalistic Settings Can Lead to Simulation Studies

**DOI:** 10.3389/fpsyg.2019.02082

**Published:** 2019-09-10

**Authors:** Jérôme Bourbousson, Mathieu Feigean, Roland Seiler

**Affiliations:** ^1^University of Nantes, Laboratory “Movement, Interactions, Performance” (EA 4334), Faculty of Sport Sciences, Nantes, France; ^2^Institute of Sport Science, University of Bern, Bern, Switzerland

**Keywords:** team coordination, shared understanding, naturalistic investigation, situated cognition, video-based field study, multi-agent system

Sport teams spend a lot of time and money to compile the best team in order to increase the chance to win. In this light, Lopez et al. (2017) analyzed how often the best team won across different team sports. Interestingly, they showed that team successes (e.g., in baseball, ice-hockey) were very little explained through team intrinsic value or potential alone. Such results came corroborate Eccles and Tenenbaum claim, when suggesting that an expert team is more than a team of experts (Eccles and Tenenbaum, 2004). Research on team sports, especially when aiming to understand team performance, has thus attempted to overlook the focus on intrinsic value of sport teams (as captured by individual talents' level), because of not accounting for “team togetherness” enough.

Historically, improving team training and team building has been targeted by the research, mainly driven by group dynamics constructs such as cohesion, leadership, and collective efficacy that were selected to investigate team togetherness. Other works investigated team learning practices (e.g., verbalization and debate-of-idea) as a part of the process of improving team intrinsic value during training, while moderate effects have been highlighted on effective team performance during games (Chow et al., 2007). More recently, real-time teamwork has been suggested a good candidate to explain on-the-field team successes (Eccles, 2010). Teamwork was defined as a main team process that make the team function effectively (McEwan and Beauchamp, 2014). Teamwork investigation thus promises to understand team performance variability resulting from team members' coordinated movements. In team sport, the most fruitful research on team performance in recent years addressed team coordination processes to better understand effective on-the-field teamwork (see Araújo and Bourbousson, 2016). Team coordination is defined as the process of arranging individual movement of team members into a patterned collective behavior. Team coordination implies individual players adjustments, thus needing a theory of how individual cognitions can merge and act together.

## Current Theoretical Perspectives on Team Coordination in Sport

While many theoretical framework have been initiated to study team coordination (e.g., Ethnomethodology for team communication on the field, LeCouteur and Feo, 2011 Natural Decision Making approach to team sports, Macquet, 2009), three theoretical frameworks for team coordination investigation have been identified as the most fruitful to date, which are the social-cognitive-, the ecological dynamics-, and the enactivist- approaches to team coordination (see Araújo and Bourbousson, 2016). In short, the social-cognitive framework considers team coordination unfold in real-time thanks to shared knowledge in teams. Based on the assumption of human as information processors, it describes how shared knowledge can be represented in groups of coordinating humans (Eccles, 2016). The ecological dynamics framework considers team coordination as occurring through affordances, in accordance with an ecological view of human cognition and perception. In this view, team coordination depends on the collective attunement to shared affordances founded on a prior platform of (mainly non-verbal) information exchange (Silva et al., 2013). Finally, the enactivist approach to team coordination considers cognition with respect to a phenomenological approach to humans, which assumes the sense-making process to highly contribute to the human–environment coupling. In this view, team coordination depends on how individual “own worlds” interact/interplays in the ongoing/unfolding interaction, making (partially) shared meaningful worlds in teams being a key phenomenon under study (Bourbousson et al., 2015).

## The Investigation of Naturalistic Settings: Empirical Evidences That Question Current Theories

In this field of research, which is actually not fully mature, some paradoxes remain, especially in that empirical evidences obtained through naturalistic games investigation question parts of existing theories, thus calling for future lines of investigation.

### Point 1—In Theory, Expert Teams Use Implicit Coordination as a Way of Interacting

As hypothesized, expert teams use implicit coordination: team members have similar expectations about how the action should unfold, in a way that allows them to coordinate accurately without any explicit verbal communication (Blickensderfer et al., 2010).

#### Related Contradictory Evidence

In their empirical study, Lausic et al. (2009) investigated double tennis teams, as operating on the field. Collecting audio-video recording of games, analyses focused on the verbal communication, as occurring on the court (i.e., amount and content of communication). The results showed that winning teams made a significant use of verbal communication (around twice more than losing teams). They also showed that winning points were characterized by more verbal communication than losing points. In terms of communication content, effective plays were well-described by verbal chaining like: “What will you do?”/“I will do that…,” suggesting that expert teams can make extensive use of overt communication to update real-time shared understanding, in that existing shared knowledge is probably not able to ensure a sufficient team togetherness. Future research should further address the way in which overt communication could be the mark of expert teams.

### Point 2—Implicit Coordination Is Achieved When Shared Knowledge Is Made Available Within the Team

As hypothesized, the more the knowledge will be shared by every team member within the team, the more the team will coordinate effortless. In such a view, a large amount of team knowledge should be shared by a lot of team members, while a low amount of knowledge should be shared by only a part of members (Eccles and Tenenbaum, 2004).

#### Related Contradictory Evidence

In their empirical study, Bourbousson and colleagues investigated high-level basketball naturalistic game (Bourbousson et al., 2011). Collecting audio-video recording and related verbalization data from every implied player, analyses were focused on eliciting the knowledge mobilized by players at every instant of the activity, and further characterizing the amount of members sharing every identified knowledge. The results showed that many players shared only very few elements of knowledge, while the most part of this knowledge was shared by only few team members. This investigation thus suggested that sharedness within the team was patterned through “local zones of sharedness,” rather than a unique zone of exhaustive sharedness. In addition, the authors showed how existing shared knowledge evolved during the game due to changes at the individual scale (to maintain the accuracy of the knowledge across the game dynamics), so that “sharedness” should be monitored/updated online. In conclusion, sharedness was assumed to be largely complemented by on-the-field dynamical processes of sharing. Future research should further address which type of knowledge shared prior to the game can serve to enhance online building of shared understanding.

### Point 3—Team Coordination Depends On Team Synergies Emerging From Player-Player Spatiotemporal Relationships

As hypothesized, on-the-field team synergies are allowed by shared affordances being available in member-member spatiotemporal relationships, so that interpersonal coordination is mainly described as “direct” (Araújo and Davids, 2016).

#### Related Contradictory Evidence

In their empirical study, R'Kiouak et al. (2016, 2018) investigated high-level rowing teams in their naturalistic performance setting. Collecting audio-video recording and related verbalization data from each team member, analyses were focused on exploring the extent to which their synergies was obtained through direct coordination vs. coordination mediated by the boat. The results showed that expert rowing team coordination improved through members becoming aware of the boat dynamics, while reducing their direct mutual awareness. The investigation of which information teammates use to adapt and help collective behavior to emerge thus suggests expert forms of team togetherness. These forms can be built on a so-called extra-personal process (Millar et al., 2013), illustrating how teamwork can be embedded in the “dynamical environment” in which it unfolds. In such a view, shared environment, when it is dynamical enough, can serve as a glue that holds together teammates activities, making shared affordances possibly located out of member-member spatiotemporal direct relationships. Thus, future research should investigate the way in which direct forms of interpersonal coordination probably need to be complemented by indirect ones, opening avenues on identifying expert patterns of team togetherness.

### Point 4—Athletes Are Attuned to Local Information

As hypothesized, affordances rely in the player-player coordination (see above for details), making players' individual awareness to be local (i.e., aware of the nearby space). At the team-scale, the chaining of local individual awareness is then assumed to be patterned enough to allow team coordination to emerge.

#### Related Contradictory Evidence

In their empirical study, Feigean and colleagues investigated a football game naturalistic setting (Feigean et al., 2018b). Collecting audio-video recording and related verbalization data from every team member, analyses were focused on the nature of information that supported players' activity when coordinating with teammates. The results confirmed the existing local information as a support for players' real-time adjustment, but highlighted how team members were also able to be attuned to global information, such as the global spatiotemporal shape they contribute to continuously emerge. In terms of sports performance, authors suggested that the players' capability to switch between local and global modes of regulation could be one important area of expertise to be considered. Future research should thus further characterize the settings in which each kind of awareness could be fruitful for team coordination.

## Toward an Alternative Model of How Teamwork is Achieved in Naturalistic Settings?

Premises of an alternative model can be drawn to understand how teamwork is achieved in naturalistic settings. While being in their infancy and needing further theorization, following statement can serve as starting points of such an alternative view of teamwork in sport that would be congruous with empirical evidences obtained in naturalistic settings of team behavior: (i) perfect moments of shared understanding and mutual awareness in action are very scarce, so that social encounter is made of “points of connection” between teammates that are episodic, local, and indirect; (ii) coordination is local/indirect, and alternates with moments of players global awareness (called holoptism, see Feigean et al., 2018b for details); (iii) players' exhibit shared sensitivity to their common environment, so that shared environment serves as a “glue” to put various “own worlds” together and to allow for cognitive entrainment within the team; (iv) while knowledge is useful during the game, its sharedness within the team is low (mainly driven by preferential interactions), the dynamics of knowledge's updating probably mattering more than the pool of knowledge shared prior to the game; (v) overt verbal communication is needed, even in expert teams, because shared understanding achievement within a team calls for online updating. Taken together, these statements open avenues for research that should be challenged in the future.

## Advancing the Research

From the current state of the research and its related paradoxes, we had identified the nature of the regulation performed by team members in real time as the major gap in current teamwork research (Bourbousson and Fortes-Bourbousson, 2016). This gap reveals that (i) the study of team performance “inputs” is far not enough to understand how team togetherness matters in explaining team successes; (ii) the description at a behavioral level of how team coordination is formed, stabilized, and destroyed was far more developed than the description of how individuals live their own interactions and regulate their teamwork in real time in relation to what they perceive as the team's behavioral needs. Thus, our purpose is to defend the individual regulation performed by members in the real-time of their spatiotemporal team coordination as a promising way of advancing the research in next years. Interestingly, a reorganization of the research in such a direction could help develop existing but few-developed alternative theoretical frameworks (i.e., not only the three main identified above), as are the ones that focalize on how each individual faces the complexity of team behavior settings (e.g., Natural Decision Making approach to team sports, Macquet, 2009; Bossard and Kermarrec, 2011) or those concerned with on-the-field social interaction (e.g., Ethnomethodology for team communication on the field, LeCouteur and Feo, 2011). The following section illustrates an innovative way of advancing the research in this line. It voluntarily breaks the codes in the field, because aiming to be enactivist while not accounting for lived experience and not being conducted with real-world sport settings. It thus will illustrate how filling current gaps in real-time teamwork research could call for innovative options.

### Feigean et al. (2018a): An Innovative Way of Advancing the Research

As a starting point, Feigean et al. considered the panel of informational resources shown to drive players on-the-field adjustments when coordinating together (see Feigean et al., 2018b for details). Instances of local informational resources can support players' activities when focalizing their perception on the ball area where the current play is unfolding, on the movement of a single player, or when exhibiting comprehensive awareness of the nearby space when looking at all proximal surrounding behaviors. Such modalities of adjustment were considered local since players do not grasp any configuration of play or multiplayer structure. In contrast, players were also able to grasp some global configurations of play, as allowed by grasping the dynamics of the game from a bird's eye viewpoint. It can occur when a player perceives multiplayers' spatiotemporal shape or when he grasps density of a given space (e.g., free space). In this mode, players could move where the density was low or attempted to avoid overcrowding an area.

Together, these modes were called individual adjustment modalities when contributing to collective behavior, and a recent study addressed the way in which we were able to capture their correlates in terms of emerging patterned collective behavior (Feigean et al., 2018a). To this end, local and global resources have been converted in two specific adjustment modalities, and converted into a simulation model of two football teams. Such a multi-agent system model has been built to be credible in terms of spatiotemporal features, and was dedicated to analyse how the collective behavior evolved, depending on both given individual adjustments modalities. When running the model, the collective behavior had specific properties, with respect for each of the local/global mode implemented as the agent adjustment modality. Such behavioral properties expressed in a combination of several metrics' values (e.g., in terms of dispersion, density, geometrical center position, etc.).

The results showed two typical team behaviors, called condensed and deployed behavior (Feigean et al., 2018a). Condensed behavior was mainly characterized by a small surface area. It was especially shaped as a vertical rectangle with width largely smaller than the length. Interestingly, configuration of condensed collective behavior was shown to be significantly the mark of a local adjustment modality. This highlights how agents being locally coupled led to an increase of the team density in a given part of the field. Obtained through a player by player effect, local couplings thus gave higher score of density in only a part of the field.

Deployed behavior was accounted by a large surface area. As described by the authors, this surface area was shaped as a horizontal rectangle with a width largely higher than the length. In such a case, stretch index was high too, what accounted for players being far from the centroid of the team. Interestingly, configuration of deployed collective behavior was shown to be significantly the mark of global adjustment modality. This shows how agents being globally coupled led to an increase of the free spaces over the field. Agents attempted to move where the density was low or attempting to avoid overcrowding an area and thus maintaining low density in any space. Taken together, the results evidenced relationships between condensed or deployed collective behavior and the local or global individual adjustment modalities, respectively. In authors' mind, the relevance and general balance allowed through a global-mode of collective adaptation should allow for better team functional exploration in complex settings in which many unexpected events can occur in any area of a wide space or by any member of a large team.

## To not Conclude…

Research gaps, as suggested above, will probably require innovative ways of conducting research. The above study conducted by Feigean et al. (2018a) illustrated how hypotheses obtained from real-time naturalistic team coordination studies can be heuristically advanced, while being investigated out of real-world sport settings. Multi-agents systems and simulation studies probably affords powerful methods to increase the knowledge about emergence in team behaviors.

## Author Contributions

JB build the rationale of the opinion and wrote first half of the manuscript. MF wrote second half of the manuscript. RS contributed to review the complete manuscript. All authors contributed to the empirical study cited in the perspectives-section.

### Conflict of Interest Statement

The authors declare that the research was conducted in the absence of any commercial or financial relationships that could be construed as a potential conflict of interest.
